# Qualitative insights into employment challenges faced by cancer patients and caregivers in a limited-resource country

**DOI:** 10.3389/fpsyg.2025.1714566

**Published:** 2026-01-13

**Authors:** Amal Al-Omari, Razan Mansour, Bayan Altalla', Hikmat Abdel-Razeq, Omar Shamieh, Akram Al-Ibraheem, Maysa Al-Hussaini, Nour Obeidat, Nisreen Qatamish, Rana Ghafary, Asem H. Mansour

**Affiliations:** 1 Office of Scientific Affairs and Research, King Hussein Cancer Center, Amman, Jordan; 2 Department of Internal Medicine, University of Kansas Medical Center, Kansas City, KS, United States; 3 Department of Internal Medicine, King Hussein Cancer Center, Amman, Jordan; 4 Department of Palliative Medicine, King Hussein Cancer Center, Amman, Jordan; 5 Nuclear Medicine Department, King Hussein Cancer Center, Amman, Jordan; 6 Cell Therapy and Applied Genomics Department, King Hussein Cancer Center, Amman, Jordan; 7 Cancer Control Office, King Hussein Cancer Center, Amman, Jordan; 8 King Hussein Cancer Foundation, Amman, Jordan; 9 Diagnostic Radiology Department, King Hussein Cancer Center, Amman, Jordan

**Keywords:** cancer, cancer caregivers, employment challenges, job security, LMIC

## Abstract

**Background:**

Cancer significantly disrupts employment, particularly in low- and middle-income countries (LMICs) where labor protections and workplace accommodations are limited. Evidence from the Middle East and North Africa (MENA) region remains scarce, and no prior qualitative research in Jordan has examined how cancer affects employment for both patients and caregivers. This study explores the employment experiences of individuals affected by cancer in Jordan and identifies systemic, workplace, and interpersonal factors shaping work participation.

**Methods:**

A qualitative descriptive design was used. Thirteen semi-structured interviews were conducted in Arabic with cancer patients, survivors, and caregivers recruited purposively from the King Hussein Cancer Center. Interviews were transcribed, translated into English, and analyzed using Braun and Clarke's reflexive thematic analysis. Trustworthiness was ensured through researcher triangulation, double-translation verification, reflexive memoing, and iterative coding.

**Results:**

Five themes captured the employment challenges faced by cancer-affected households: (1) rigid and insufficient sick leave provisions that fail to accommodate the prolonged and cyclical nature of cancer treatment; (2) workplace stigma and disclosure dilemmas, contributing to concealment, marginalization, or altered workplace relationships; (3) financial toxicity driven by lost income, transportation expenses, and debt accumulation; (4) limited workplace accommodations and health–job mismatches, particularly in manual and private-sector roles; and (5) the invisible burden on caregivers, who faced employment conflicts and lacked statutory caregiver leave. These findings reveal structural gaps in Jordan's labor policies and social protection systems.

**Conclusions:**

Cancer patients and caregivers in Jordan experience substantial employment vulnerability shaped by inadequate leave policies, stigma, financial strain, and a lack of accommodations. Policy reforms: including extended medical leave, anti-discrimination protections, statutory caregiver leave, flexible work arrangements, and strengthened income-support mechanisms, are essential to improving the employment sustainability and wellbeing of cancer-affected households.

## Introduction

Cancer survivorship has become a critical global public health concern, as advances in early detection and treatment have significantly improved survival rates across many cancer types (Mehnert, 2011). Returning to work after a cancer diagnosis remains a profound challenge, influencing patients' physical health, psychological wellbeing, and socioeconomic stability (Stergiou-Kita et al., 2016). Work is universally regarded as a cornerstone of personal identity, social inclusion, and financial stability (Taskila and Lindbohm, 2007; Sesto et al., 2022). However, cancer survivors often face job loss, reduced working hours, and workplace discrimination (Taskila and Lindbohm, 2007; Sesto et al., 2022). Employment disruption among cancer survivors significantly impacts psychological wellbeing, identity, and social integration. Survivors often face anxiety, reduced self-confidence, and social isolation, exacerbated by workplace stigma and fear of job loss (Shim et al., 2021). Inability to return to work undermines role functioning and sense of normalcy, while employment resumption supports recovery and self-worth (Kinaci Gümüşçubuk and Çalik Başaran, 2024; Kane et al., 2020). Working survivors report better mental health outcomes than non-working peers, highlighting employment as a protective factor (Lieb et al., 2022). Supportive workplaces can mitigate stigma and enhance psychological resilience (Su et al., 2024).

While high-income countries have implemented policies and workplace accommodations to address these issues, disparities remain (Mehnert, 2011; Alleaume et al., 2020). The challenges are often more pronounced in low- and middle-income countries (LMICs), where limited labor protections, informal employment structures, and insufficient social support systems exacerbate the vulnerability of cancer patients (dos Anjos et al., 2025). In the Middle East and North Africa (MENA) region, and specifically Jordan, these difficulties are compounded by high unemployment rates, scarce workplace accommodations for chronic illnesses, and cultural expectations surrounding caregiving and gender roles (Mansour et al., 2024).

International research consistently highlights the impact of cancer on employment (De Boer et al., 2009; Yoo et al., 2013; Stone et al., 2017). A meta-analysis has shown that cancer survivors are ~1.4 times more likely to be unemployed compared with the general population (De Boer et al., 2009). Work disruptions differ by cancer type, treatment, and sociodemographic characteristics, with breast, lung, and hematologic cancers often linked to longer work absences and reduced productivity (Lyons et al., 2022; Mehnert et al., 2013). Moreover, caregivers; whether parents of pediatric patients or family members of adults, face significant occupational strain, including absenteeism, reduced performance, and in some cases withdrawal from the labor force (Kent et al., 2016; Bevans and Sternberg, 2012). Despite this growing body of global evidence, research in the MENA region remains scarce and fragmented, with most studies focusing on clinical or psychosocial outcomes rather than employment trajectories (Al-Omari et al., 2022). Importantly, recent reviews note a near-complete absence of qualitative studies exploring cancer-related employment challenges in Arab countries (Kemp et al., 2025). No prior research in Jordan has examined how workplace policies, cultural norms, and caregiving demands shape employment experiences. Additionally, no existing studies integrate both patient and caregiver perspectives, despite their interlinked roles in navigating cancer and work.

This study aims to address this gap by exploring the perspectives of patients with cancer and their caregivers regarding employment in Jordan. By employing a qualitative design grounded in in-depth interviews, the research seeks to generate insights into the complex interplay of illness, caregiving responsibilities, and work. Findings from this study will provide evidence to inform workplace practices and policymaking to better support cancer survivors and their families within Jordan and the wider region.

## Methods

A qualitative descriptive design was used to explore the employment experiences of cancer patients, survivors, and caregivers in Jordan. This design is appropriate for applied health research aimed at generating clear, practice-oriented insights grounded in participants' accounts (Bradshaw et al., 2017). Data were analyzed using Braun and Clarke's reflexive thematic analysis, which offers a systematic yet flexible approach to identifying and interpreting patterns across qualitative datasets (Braun and Clarke, 2023).

## Participant recruitment and sampling

Participants were purposively recruited through the King Hussein Cancer Center (KHCC) to ensure diversity in gender, age, cancer type, treatment stage, employment sector, and caregiving responsibilities. Eligible participants were adults aged 18 years or older who had been diagnosed with cancer or were serving as the primary caregiver of a patient with cancer, and who had been employed at any point during or after the cancer trajectory, enabling them to speak directly about work-related experiences. Individuals were excluded if they had no employment experience during or after the cancer trajectory, were unable to provide informed consent, or were caregivers not involved in employment-related decision-making for the patient. In cases where a patient was medically or cognitively unable to participate, their primary caregiver was interviewed on their behalf. A total of 13 participants were enrolled, including cancer survivors, patients undergoing active treatment, and caregivers of both pediatric and adult patients. Recruitment continued until the research team determined that thematic sufficiency had been reached. Following Hennink and Kaiser (Guest et al., 2020; Hennink and Kaiser, 2022), we assessed saturation by monitoring whether new interviews produced conceptually new information. After approximately the 11th interview, no new codes emerged, and the final two interviews confirmed code stability rather than expansion.

## Data collection

Data were collected through in-depth, semi-structured interviews conducted in Arabic between December 2024 and March 2025. The interview guide included open-ended questions and follow-up prompts exploring workplace interactions, disclosure decisions, sick leave experiences, financial challenges, and caregiving demands. The guide was pilot tested with two individuals and refined for clarity and relevance.

Interviews lasted between 35 and 60 min, were conducted in private rooms at KHCC, and audio-recorded with participants' consent. Recordings were transcribed verbatim in Arabic. A professional translator produced English translations, which were then reviewed and verified by bilingual researchers to ensure accuracy and retention of meaning. During translation, bilingual researchers examined idiomatic expressions, cultural metaphors, honorifics, and context-specific phrasing to ensure psychological meaning was preserved. Any ambiguous expressions were discussed in analytic meetings, and decisions were documented through reflexive memoing to ensure transparency and cultural fidelity.

## Ethical approval and data management

The study received ethical approval from the KHCC Institutional Review Board (IRB approval number 24KHCC218). All participants provided written informed consent and were informed of their right to withdraw at any time without consequence. Audio files and transcripts were stored on encrypted, password-protected servers accessible only to the research team. All transcripts were anonymized, and identifying information was removed to protect confidentiality.

## Data analysis

Data were analyzed using Braun and Clarke's six-step reflexive thematic analysis: (1) familiarization with the data, (2) generation of initial codes, (3) development of candidate themes, (4) review and refinement of themes, (5) definition and naming of themes, and (6) production of the final report (Braun and Clarke, 2023).

## Results

A total of 13 participants were included in this study, comprising 10 patients diagnosed with cancer and 3 caregivers providing primary care to a family member with cancer. Participants ranged in age from 14 to 63 years, with representation from both sexes and various employment sectors, including manual labor, transportation, academia, and public service. Caregivers included parents and siblings who had either reduced their working hours or left employment entirely to provide care. Patients' characteristics are shown in Table 1.

**Table 1:** Participant characteristics (*N* = 13).

**Participant ID**	**Role**	**Patient's age**	**Patient's gender**	**Patient's diagnosis**	**Participant's employment sector**
P1	Patient	22	Male	Sarcoma	Private sector
P2	Patient	25	Male	Brain cancer	Private sector
P3	Patient	63	Male	Colon cancer	Public sector
P4	Patient	25	Female	Lymphoma	Private sector
P5	Patient	25	Male	Brain cancer	Private sector
P6	Caregiver	36	Female	Sarcoma	Public sector
P7	Caregiver	14	Male	Brain cancer	Private sector
P8	Patient	41	Female	Bladder cancer	Public sector
P9	Patient	36	Male	Leukemia	Private sector
P10	Patient	49	Male	Pancreatic cancer	Private sector
P11	Patient	62	Male	Central nervous system cancer	Private sector
P12	Patient	41	Male	Head and neck cancer	Private sector
P13	Caregiver	54	Male	Leukemia	Not reported

## Analytic overview

**Figure 1:**
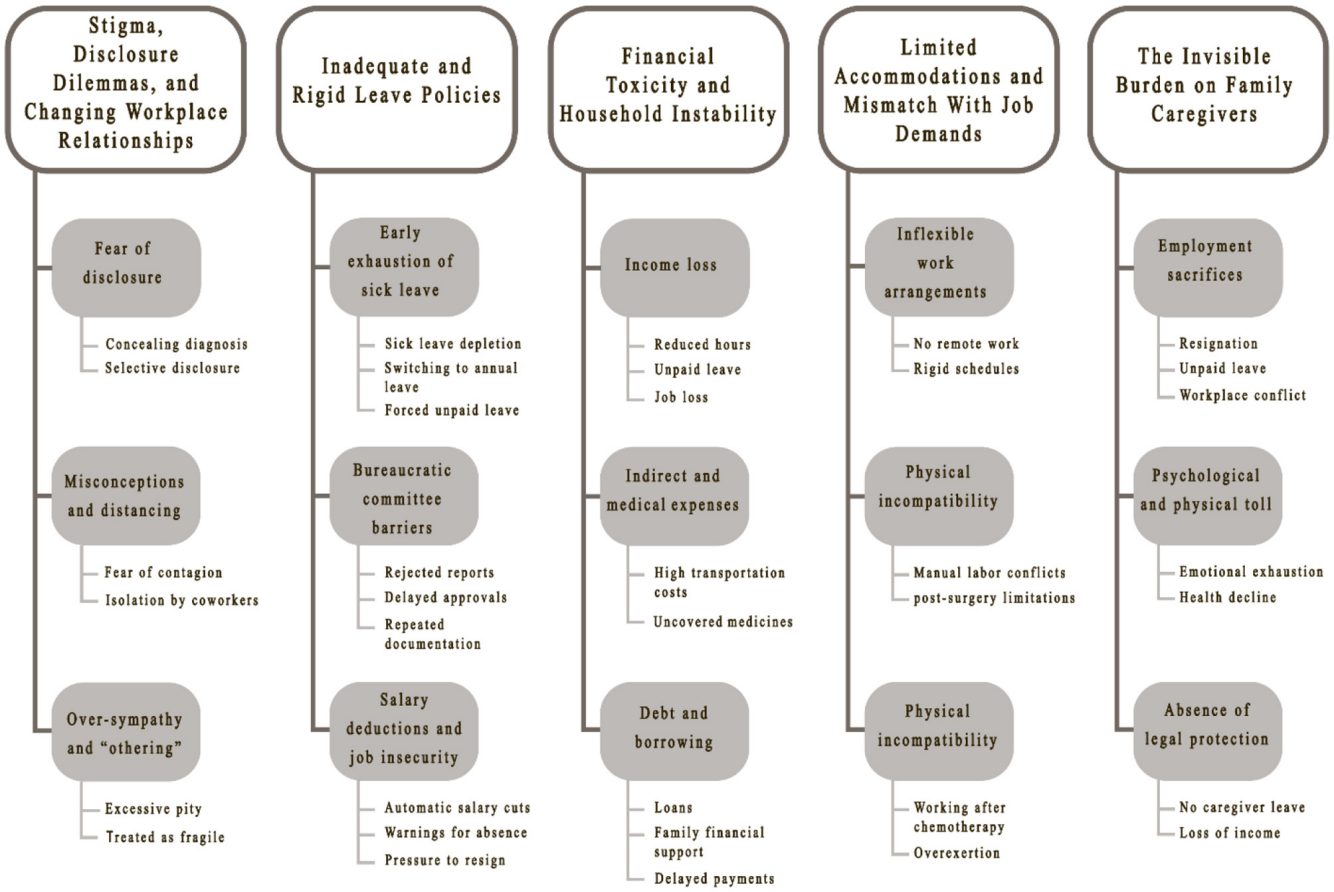
Coding framework (themes, subthemes, and representative codes).

Reflexive thematic analysis produced 144 initial codes, which were systematically reviewed, compared across interviews, and refined into conceptual categories through iterative abstraction. Five overarching themes were generated, each containing several subthemes that reflect patient and caregiver experiences with employment during cancer. The themes demonstrate the interaction of structural, organizational, and interpersonal factors shaping employment vulnerability. To enhance transparency, the coding structure is summarized in Figure 1.

## Thematic findings

**Figure 2:**
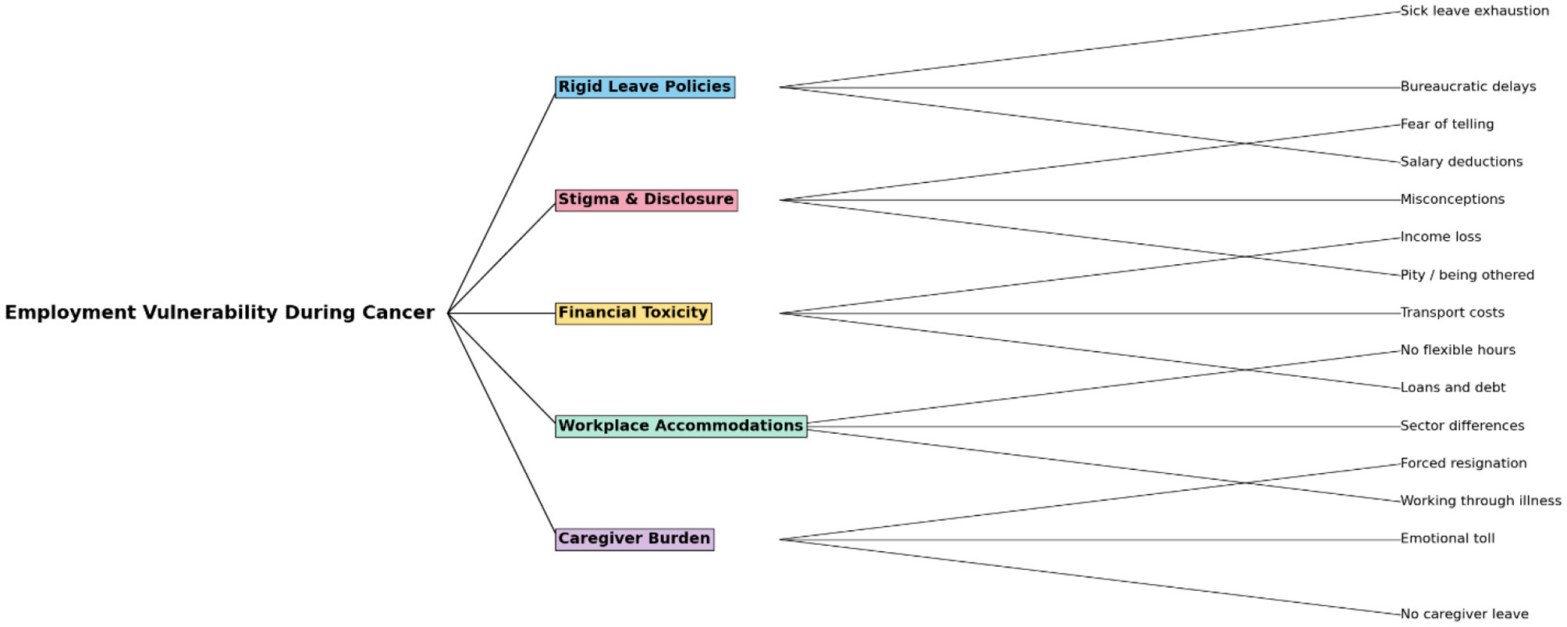
Network graph representing the employment vulnerability during cancer.

Thematic analysis revealed five linked themes that reflect the challenges faced by cancer patients and their caregivers in maintaining employment throughout the illness journey. The themes illustrate the cumulative effects of rigid policies, workplace stigma, economic strain, and lack of formal protection, framed through the lived experiences of participants. The thematic map shown in the Figure 2 conceptualizes “Employment Vulnerability during Cancer” as the central phenomenon influenced by five intersecting domains.

## Theme 1: inadequate and rigid leave policies jeopardizing job retention

Participants consistently described how sick leave entitlements were insufficient in the context of cancer treatment, which often requires extended or recurring absences from work. Several reported exhausting their entire leave allowance soon after diagnosis. One patient shared, “*I used all my sick leave in the first month. After that, every chemotherapy session I had to negotiate time off, and then they started deducting from my salary (P10, male, 49)”*. Processes for extending leave were perceived as bureaucratic and slow, often leading to income loss during the waiting period. As one participant explained, “*The committee kept returning my papers for more signatures, and until they approved it, my salary went down to 160 dinars (P13 caregiver, male, 54)”*. Participants described needing to provide repeated documentation, secure multiple approvals, and wait through lengthy reviews. While patients struggled with exhausting limited sick leave early in treatment, caregivers faced similar bureaucratic hurdles when requesting time off to accompany loved ones. Several caregivers reported using personal sick leave or annual leave, and when exhausted, were forced into unpaid leave or resignation.

In especially distressing cases, terminally ill individuals were required to appear in person before medical committees to validate their condition. Caregivers highlighted how such requirements added to emotional distress and logistical difficulties, especially when patients were physically weakened. Across accounts, leave policies were perceived as misaligned with the clinical reality of cancer, lacking both flexibility and compassion.

## Theme 2: stigma, disclosure dilemmas, and shifting workplace relationships

The decision to disclose a cancer diagnosis at work was described as emotionally fraught and strategically calculated. Fearing judgment or job loss, many initially kept their illness private. One participant shared, “*I hid my illness in the beginning because I feared they would stop giving me work. (P12, male, 41)”*.

For those who did disclose, reactions from colleagues were mixed. Some faced subtle or overt distancing, often rooted in misinformation or anxiety about productivity. One participant observed, “*Some colleagues avoided me because they thought cancer was contagious (P2, male, 25)”*. Others described being met with excessive sympathy that made them feel infantilized or singled out as fragile. These responses often disrupted professional relationships and contributed to a loss of workplace confidence. Patients noted how the combination of pity, avoidance, or overprotection altered their role and social integration at work. The impact of disclosure extended beyond individual interactions, often reshaping one's entire workplace experience.

## Theme 3: financial toxicity and the erosion of household security

Nearly all participants, both patients and caregivers, described intense financial strain caused by cancer. Income loss due to unpaid leave or reduced work hours intersected with rising medical costs and logistical expenses, such as transportation. One patient explained, “*Each visit from my home costs me around 50 dinars. That's just to get to the hospital (P12, male, 56)”*.

To survive, families often turned to borrowing and informal support networks. As one participant put it, “*I borrowed more than 20,000 dinars from the bank and relatives just to keep up with expenses (P10, male, 49).”* The financial impact extended beyond direct treatment costs, affecting household budgets, debt levels, and savings.

For caregivers, the financial toll stemmed primarily from lost income due to job interruption and caregiving duties. Unlike patients, they were often ineligible for direct financial support and faced dual burdens of supporting treatment costs while losing income. One caregiver noted, “I had to reduce my work hours, but no one helps the caregivers financially” (P6, caregiver, female, 36).

## Theme 4: limited workplace accommodations and health–job mismatch

Workplace accommodation was described as inconsistent and often dependent on the nature of the job and the disposition of individual employers. Participants in office-based or academic roles sometimes received modified tasks or flexible schedules. However, those in physically demanding or private-sector jobs frequently receive no such support.

One patient, employed in transportation, noted, “*After kidney surgery and chemo, I couldn't even climb into the truck (P11, male, 62)”*. Despite serious limitations, some participants continued working during treatment, driven by fear of job loss or income disruption. As one patient put it, “*went to work after chemotherapy many times. I was tired, dizzy, but I had no choice (P2, male, 25)”*.

These accounts highlighted a mismatch between job demands and health status, particularly in labor-intensive roles. The lack of formal mechanisms to adjust work responsibilities contributed to health deterioration and forced presenteeism among patients.

## Theme 5: the invisible burden on family caregivers in an unsupportive legal environment

In contrast to patients, caregivers were neither the direct recipients of medical leave nor recognized formally by their employers, placing them in a uniquely precarious position. The emotional and physical toll was compounded by legal invisibility. Many used their own sick leave, took unpaid leave, or ultimately left their jobs to care for loved ones. One caregiver explained, “*I ran out of leave. I started taking unpaid days and sometimes claimed I was sick. In the end, I resigned because I couldn't balance both (P6 caregiver, female, 36)”*.

In addition to the economic consequences, caregivers experienced psychological distress and, in some cases, deterioration in their own health due to the prolonged demands of caregiving. The lack of institutional recognition for their role deepened the sense of exclusion. As one caregiver stated, ”*There is no leave for caregivers. Families are invisible in the system. (P13 caregiver, male, 54)”*.

These experiences revealed a critical gap in employment protections for family members supporting patients, underscoring the emotional and systemic costs of caregiving within an unsupportive legal framework.

## Summary of findings

Together, these five themes depict a landscape in which cancer patients and their families struggle to balance employment, health, and caregiving under rigid institutional and legal constraints. Inadequate leave policies, workplace stigma, financial precarity, insufficient accommodation, and lack of legal protections collectively undermined job retention and household stability. The findings underscore the urgent need for systemic policy reform to address the multidimensional vulnerabilities faced by cancer-affected households.

## Discussion

This study provides the first qualitative exploration of employment challenges faced by patients with cancer and their caregivers in Jordan. Using reflexive thematic analysis, the findings reveal a multilayered landscape of vulnerability shaped by structural, organizational, and interpersonal factors. Although similar employment barriers have been documented among cancer survivors globally, this study highlights how these challenges are intensified in limited-resource settings where labor protections, workplace accommodations, and caregiver support policies remain underdeveloped. These findings resonate with international literature documenting that cancer survivors are more likely to experience unemployment, job loss, and financial insecurity compared with the general population (De Boer et al., 2009; Yoo et al., 2013; Stone et al., 2017), but they also emphasize the unique vulnerabilities shaped by Jordan's legal and sociocultural context.

In Jordan, the Labor Law No. 8 of 1996 grants employees a maximum of 14 days of paid sick leave per year (extendable to 28 days with a medical committee report), but this allowance is clearly insufficient for managing chronic or cyclical conditions like cancer (World Intellectual Property Organization, 1996). Notably, the law does not recognize caregiver leave, and no statutory framework exists to protect family members who must reduce work or resign to provide care. Few studies in the MENA region have explored cancer-related employment, yet the available evidence parallels our findings (Al-Omari et al., 2022). Research from Lebanon and Egypt reports similar challenges around stigma, limited workplace accommodations, and the financial vulnerability of families affected by cancer (Abu-Saad Huijer et al., 2013; Gany et al., 2020). Studies from Gulf countries highlight gaps in labor protections for chronic illnesses, underscoring the need for region-specific policies (Alaloul et al., 2025; Mula-Hussain et al., 2013). These regional parallels reinforce the urgency of addressing employment-related psychosocial burdens within Middle Eastern health and labor systems.

One of the most consistent findings was the inadequacy of sick leave provisions. Participants frequently reported exhausting the legally mandated allowance of 14–28 days within the first weeks of treatment, forcing them into unpaid leave or resignation (World Intellectual Property Organization, 1996). Similar patterns have been documented in other LMICs, where labor laws are structured around short-term illness and fail to accommodate the extended recovery periods associated with cancer (dos Anjos et al., 2025). In Jordan, rigid administrative requirements, such as repeated medical committee evaluations, further compounded challenges by delaying leave approval, disrupting income continuity, and creating additional emotional and logistical strain (World Intellectual Property Organization, 1996). These bureaucratic hurdles not only jeopardize job retention but may also undermine treatment adherence. By contrast, in high-income countries, extended sick leave and job-protected absence schemes are standard. In the United States, for example, the Family and Medical Leave Act (FMLA) guarantees eligible employees up to 12 weeks of unpaid, job-protected leave for serious health conditions, while the Americans with Disabilities Act (ADA) prohibits discrimination and requires employers to provide reasonable accommodations such as flexible hours or modified duties (Golden, 1991; Rumrill, 1999).

Fear of disclosure and workplace stigma further compounded participants' sense of job insecurity. Some chose not to disclose their diagnosis to employers, while others described discriminatory treatment once their illness became known. These accounts are consistent with evidence from the region internationally, which shows that stigma, often driven by misconceptions about contagion, diminished productivity, or inevitable decline, remains a major barrier to sustained employment among cancer survivors (Hallgren et al., 2023; Kong et al., 2021). Internationally, legal frameworks such as the Americans with Disabilities Act in the United States and the Equality Act in the United Kingdom prohibit dismissal and mandate reasonable workplace accommodations for cancer survivors (Golden, 1991; Humphreys, 2010). However, even robust legal frameworks like the U.S. Family and Medical Leave Act (FMLA) and Americans with Disabilities Act (ADA) reveal practical limitations: FMLA provides unpaid leave and excludes nearly 40% of U.S. workers, making it inaccessible to many low-income households (Davison and Blackburn, 2023). Similarly, while the ADA mandates reasonable accommodations, it does not guarantee paid leave and varies in enforcement depending on employer capacity (Davison and Blackburn, 2023; Romig and Bryant, 2021). These examples highlight the need to adapt legal models to Jordan's context, which lacks such protections, leaving employees vulnerable to dismissal or subtle pressures to resign, as described by our participants. Introducing anti-discrimination legislation that explicitly covers chronic illness, including cancer, would represent an important step toward safeguarding workers' rights.

Financial strain emerged as another cross-cutting theme, with patients describing a cycle of lost wages, reduced work hours, and mounting out-of-pocket expenses. Although Jordan's healthcare system subsidizes many cancer treatments, ancillary costs such as transportation and medications were often uncovered, and gaps in employment left patients without critical income or health insurance continuity. These findings mirror evidence from both LMICs and high-income settings that financial toxicity is a common consequence of cancer (Kemp et al., 2021; Johnston et al., 2023). Yet, unlike many high-income countries, where partial wage replacement or disability benefits are available (Rumrill, 1999; Barrett, 2022). Jordan provides only modest incapacity pensions, leaving many families without adequate protection. Participants' testimonies illustrate how such economic pressures compound the psychological and physical burden of cancer.

The experiences of caregivers add an additional dimension to these challenges. Family members frequently reported conflicts with their employers, exhaustion of leave entitlements, and in some cases resignation from work to provide care. Jordanian law does not currently provide specific leave for caregiving responsibilities, leaving families to rely on employer goodwill. The absence of such provisions in Jordan not only jeopardizes household financial security but also threatens long-term career trajectories and pension contributions for caregivers.

By situating these findings within international experience, clear policy implications emerge. Legal reforms are urgently needed to extend sick leave entitlements, introduce anti-discrimination protections, and establish caregiver leave as a statutory right. Embedding flexible work arrangements and reintegration programs into labor policies would also help survivors remain active in the workforce, aligning Jordan's practices with global standards. Beyond the national level, these recommendations are consistent with Jordan's obligations as a signatory to the United Nations Convention on the Rights of Persons with Disabilities and contribute directly to achieving the Sustainable Development Goals, particularly SDG 3 on health and wellbeing and SDG 8 on decent work and economic growth (Ferranti, 2018; ILO, 2024).

## Implications for policy and practice

Collectively, these findings underscore the need for comprehensive policy reform to protect the employment and socioeconomic stability of cancer-affected individuals in Jordan. Medical leave provisions require extension and restructuring to accommodate the long-term and cyclical nature of cancer treatment, as current limits are incompatible with clinical realities. Anti-discrimination protections must also be introduced to explicitly include cancer and other chronic illnesses, thereby safeguarding employees from dismissal or subtle forms of exclusion following diagnosis. In parallel, establishing statutory caregiver leave would acknowledge the essential role family caregivers play in health systems and protect them from employment loss and financial instability. Workplaces should additionally adopt formal accommodation policies, such as flexible scheduling, modified duties, or remote work options, to support continued employment during treatment and recovery. Finally, strengthening social protection mechanisms through temporary wage replacement, expanded disability benefits, or other financial support programs is critical to mitigating the substantial financial toxicity experienced by patients and caregivers. Together, these measures represent key steps toward creating an equitable and supportive employment environment for individuals navigating cancer.

Implementation in Jordan must also consider economic constraints, such as a persistently high unemployment rate (~25%) and limited fiscal space due to rising public debt (Al-Ajlouni, 2021). For reform to be realistic and effective, gradual policy rollout, employer incentives, and cross-sector partnerships may be needed. Still, formalizing workplace accommodation (e.g., flexible scheduling, remote work) and expanding income protection (e.g., wage replacement or temporary disability support) remain essential to reducing employment-related distress for cancer-affected households.

## Strengths and limitations

This study offers rich insights into cancer-related employment challenges from both patient and caregiver perspectives, an area previously undocumented in Jordan and the wider MENA region. The use of reflexive thematic analysis, triangulation among trained researchers, and rigorous data verification procedures enhances the credibility of the findings.

However, several limitations must be acknowledged. The purposive sample of 13 participants limits transferability, although depth of insight rather than breadth is the aim of qualitative inquiry. As participants were recruited from a single major cancer center in Jordan, the experiences of individuals treated in rural areas or non-specialized facilities may differ, potentially limiting the generalizability of findings. Data collection relied on self-reported accounts, which may be influenced by recall or social desirability bias. Finally, the study did not include employers or policymakers, whose perspectives could provide additional context regarding institutional constraints and opportunities for reform.

Future research should explore these viewpoints, incorporate larger and more diverse samples, and consider comparative analyses across Arab countries to inform regionally relevant policy development.

## Conclusions

In conclusion, this study demonstrates that cancer patients and their caregivers in Jordan face multiple employment challenges driven by inadequate labor protections, workplace stigma, and lack of supportive policies. Without reform, these barriers will continue to undermine both individual livelihoods and broader social and economic participation. Updating Jordan's legal and policy frameworks to protect the employment rights of cancer-affected workers and caregivers is not only a matter of social justice but also an economic imperative.

## Data Availability

The raw data supporting the conclusions of this article will be made available by the authors, without undue reservation.
