# Supplementary material for: Qualitative insights into employment challenges faced by cancer patients and caregivers in a limited-resource country
Source: Front Psychol. 2026 Jan 13;16:1714566. doi: 10.3389/fpsyg.2025.1714566 (PMC12835211; doi:10.3389/fpsyg.2025.1714566)
Supplement: Supplementary file 1 [file Data_Sheet_1.docx]

**Appendix:**

**Interview Guide**

**Workers Providing Care for a Family Member with Cancer**

The study aims to discuss the challenges facing the employment of patients with cancer, identify legal shortcomings and potential gaps in the current legal frameworks, and propose recommendations and alternative policies to enhance legal protection in the Jordanian labor market. This includes support mechanisms for both workers who are patients with cancer and caregivers of family members or first-degree relatives with cancer.

**Axis 1: Leave (Paid and Unpaid)**

- Do you consider the duration of sick/annual leave sufficient to meet your needs for providing care to a family member with cancer? Please explain.
- How are requests for extending annual or additional leave for the purpose of caring for a family member with cancer handled?
- If you experience physical or psychological exhaustion, are you able to obtain sick leave? What are the conditions required for this?
- How are requests for unpaid leave from caregivers to care for patients with cancer handled? What challenges do you face if you are forced to take long-term unpaid leave?
- Do companies or institutions provide exceptional or humanitarian leave due to your caregiving responsibilities for a family member with cancer? How are these leaves organized, if they exist?
- What role do internal workplace policies play in supporting you as a caregiver?
- From your perspective, how do leave policies differ between the public and private sectors regarding caregivers of family members with cancer, if any?

**Axis 2: Supportive Work Environment**

- What difficulties or challenges do you face at work during the treatment period of a family member with cancer? (Discussion points: psychological and emotional stress, job retention during treatment, balancing work and caregiving, absenteeism, lack of workplace support, impact on income, impact on career development)
- Are workplaces in Jordan adequately prepared to support workers who are caregivers for family members with cancer undergoing long-term treatments? How would you describe your experience in workplaces regarding receiving support and facilities? Can you share an experience you have had?
- Are you provided with facilities such as reduced working hours or flexible work arrangements? How does this affect your ability to provide care for a family member with cancer?
- Are there financial aids provided to you due to your caregiving responsibilities for a family member with cancer? What are the current gaps in providing financial assistance, and how can they be addressed from your perspective? (Discussion points: social security, National Aid Fund, NGOs and charities, Zakat Fund)
- How do you perceive the interaction between you and employers in terms of flexibility in the work environment? How can the role of psychological and social support programs in the workplace be enhanced to assist you at work? (Discussion points: flexible work, remote work, part-time work, flexible hours, compressed workweek, flexible year, financial assistance or special grants to cover treatment costs or unpaid leave periods)
- How does the Labor Law/Social Security Law affect your economic and social situation and that of your family? What role can stakeholders such as the Ministry of Labor, Social Security, and civil society organizations play in improving your working conditions? (Discussion points: impact on income, treatment costs, financial dependence on the family, taking on additional responsibilities, psychological effects, work challenges, health coverage, loss of skills due to work interruptions)
- What recommendations do you propose to improve the economic, social, and psychological support for caregivers of family members with cancer during and after the treatment period?
